# Sea level rise risks and societal adaptation benefits in low-lying coastal areas

**DOI:** 10.1038/s41598-022-14303-w

**Published:** 2022-06-23

**Authors:** Alexandre K. Magnan, Michael Oppenheimer, Matthias Garschagen, Maya K. Buchanan, Virginie K. E. Duvat, Donald L. Forbes, James D. Ford, Erwin Lambert, Jan Petzold, Fabrice G. Renaud, Zita Sebesvari, Roderik S. W. van de Wal, Jochen Hinkel, Hans-Otto Pörtner

**Affiliations:** 1 Institute for Sustainable Development and International Relations (IDDRI-Sciences Po), Paris, France; 2 LIENSs Laboratory UMR7266, CNRS & University of La Rochelle, La Rochelle, France; 3 Department of Geosciences and the School of Public and International Affairs, Princeton University, Princeton, NJ USA; 4 Department of Geography, Ludwig-Maximilians-Universität München (LMU), Munich, Germany; 5 Climate Central, Princeton, NJ USA; 6 Natural Resources Canada, Bedford Institute of Oceanography, Dartmouth, Canada; 7 Priestley International Centre for Climate, University of Leeds, Leeds, UK; 8 Institute for Marine and Atmospheric Research Utrecht, Utrecht University, Utrecht, The Netherlands; 9 Royal Netherland Meteorological Institute (KNMI), De Bilt, The Netherlands; 10 Center for Earth System Research and Sustainability (CEN), University of Hamburg, Hamburg, Germany; 11 School of Interdisciplinary Studies, University of Glasgow, Dumfries, UK; 12 Institute for Environment and Human Security, United Nations University, Bonn, Germany; 13 Department of Physical Geography, Utrecht University, Utrecht, The Netherlands; 14 Global Climate Forum, Berlin, Germany; 15 Albrecht Daniel Thaer-Institute and Berlin Workshop in Institutional Analysis of Social-Ecological Systems (WINS), Humboldt-University, Berlin, Germany; 16 Alfred Wegener Institute, Bremen, Germany

**Keywords:** Climate-change impacts, Climate-change adaptation, Climate-change impacts

## Abstract

Sea level rise (SLR) will increase adaptation needs along low-lying coasts worldwide. Despite centuries of experience with coastal risk, knowledge about the effectiveness and feasibility of societal adaptation on the scale required in a warmer world remains limited. This paper contrasts end-century SLR risks under two warming and two adaptation scenarios, for four coastal settlement archetypes (Urban Atoll Islands, Arctic Communities, Large Tropical Agricultural Deltas, Resource-Rich Cities). We show that adaptation will be substantially beneficial to the continued habitability of most low-lying settlements over this century, at least until the RCP8.5 median SLR level is reached. However, diverse locations worldwide will experience adaptation limits over the course of this century, indicating situations where even ambitious adaptation cannot sufficiently offset a failure to effectively mitigate greenhouse-gas emissions.

## Introduction

**Figure 1:**
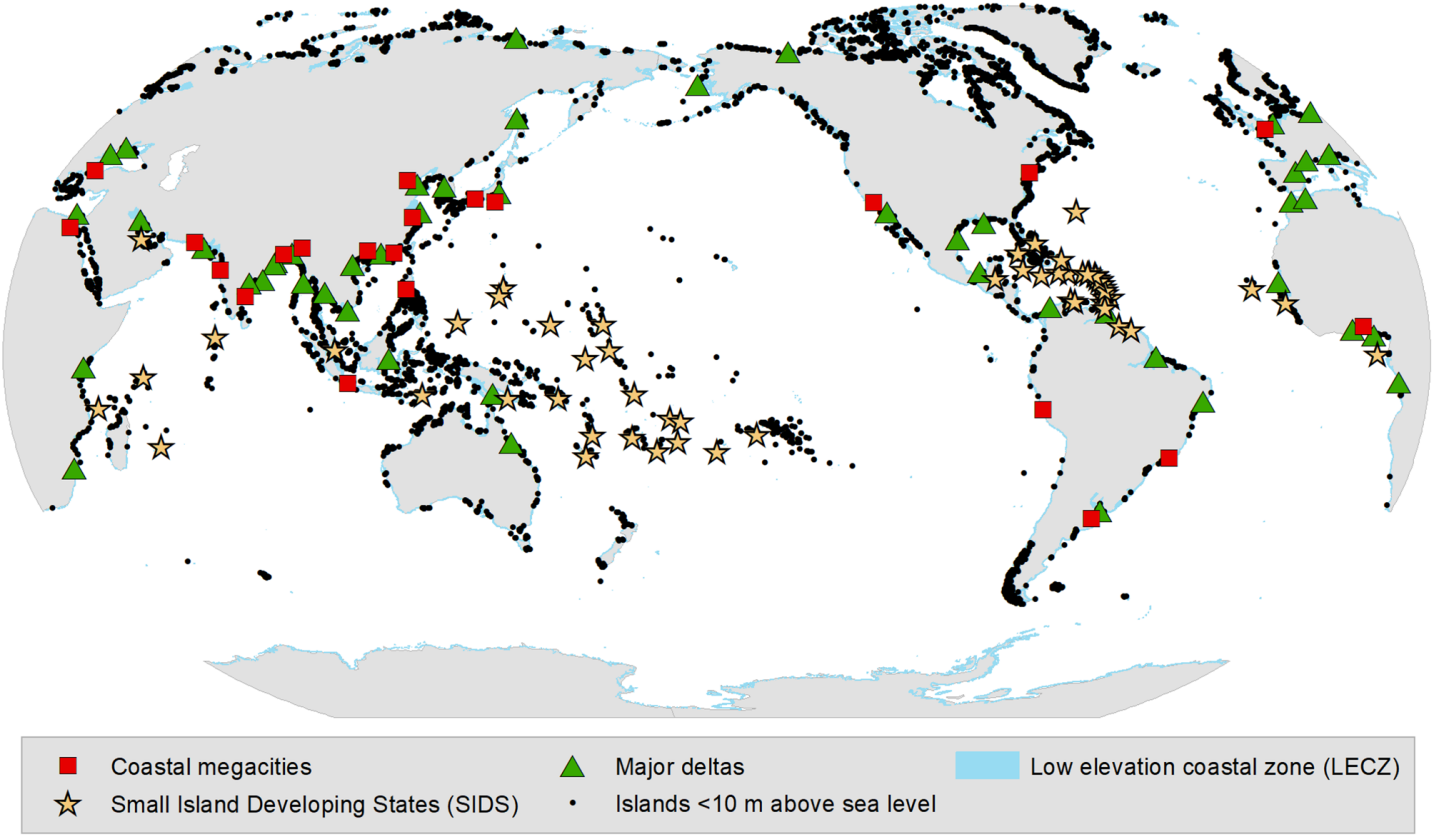
Global distribution of low-lying islands and coasts. The map shows Low Elevation Coastal Zones (coasts < 10 m above sea level; blue lines; Source: National Geophysical Data Center, NOAA, https://data.nodc.noaa.gov/cgi-bin/iso?id=gov.noaa.ngdc.mgg.dem:280), islands with a maximum elevation of 10 m above sea level (black dots), Small Island Developing States (yellow stars; Source: http://unohrlls.org/about-sids/), coastal megacities (> 10 million inhabitants, < 100 km from the coast, < 50 m above sea level; red squares), and major deltas (green triangles).

Many low-lying coastal areas face serious risks from climate change because of their modest elevation above sea level, climate-sensitive physical and ecological characteristics (e.g. coral beaches, sea ice environments), and high societal exposure and vulnerability (e.g. flood-prone high population and asset density, marine-dependent small-scale economies). The low-elevation coastal zone (LECZ) comprises continental and island areas hydrologically connected to the sea and no more than 10 m above mean sea level^1^. It includes a wide diversity of systems, from small islands to megacities, from the Tropics to the Poles, in both the Global North and the Global South (Fig. 1), currently hosting ~ 11% of the global population^2^ and generating ~ 14% of the global Gross Domestic Product^3^.

Climate change is inducing slow onset changes in the physics and chemistry of the global ocean, including acidification and warming, loss of sea ice, and sea level rise (SLR)^4^. SLR and increases in frequencies of extreme sea levels at the coast are widely considered among the highest climate priorities by policy-makers and the public worldwide^5^. Moreover, by the end of this century, marine and terrestrial coastal ecosystems will experience substantial modification (changing location and spatial contraction)^6–8^ and a loss of both functionality and biodiversity^9–11^. Even assuming that the population of the LECZ remains fixed and ignoring future changes in natural and human-made coastal protection as well as changes in the climatology of storms associated with extreme sea levels, the population living below projected annual flood levels is expected to more than double in the case of a 1 m global mean SLR^12^. Considering the potential for an additional coastal population increase, this figure is likely to be higher.

In light of these risks, societal adaptation is recognized as essential, even at lower levels of warming^4,13,14^. However, the literature on adaptation outcomes remains either too broad in scope (such as in the IPCC reports^15,16^)*,* too narrowly model-based and focused (for example, on SLR-related flooding as the only consequence and the idea of hard protection as the only response^17–19^)*,* or limited to in-depth examination of single case studies^20^, for a comprehensive analysis of required adaptation to be made. A major knowledge gap remains about the potential effectiveness of a wide range of coastal adaptation to risk reduction in the future^17,21^.

This paper addresses these gaps by focusing on the additional coastal risks induced by SLR. It presents an integrated risk and adaptation assessment that takes into account multiple natural and human drivers and impacts, as well as the potential benefits of diverse adaptation measures for risk reduction depending on coastal archetypes. It builds on the key findings of a formal expert judgment exercise to assess SLR risks to four coastal settlement archetypes (Urban Atoll Islands, Arctic Communities, Large Tropical Agricultural Deltas, and Resource-Rich Cities; see Box 1), initiated as part of the IPCC *Special Report on Ocean and Cryosphere in a Changing Climate* (SROCC)^4,17^. Risk levels are assessed for two time horizons (present-day and end of this century), two contrasting emission pathways (RCP2.6 and RCP8.5) and two alternative societal adaptation scenarios (none-to-moderate and high) (see “Methods”). This material lays the foundation for an enhanced understanding of SLR adaptation benefits (see Box 2). Our approach, while developed and tested for globally relevant coastal archetypes, also may be used for future climate adaptation research in other settings. Last, the paper examines the implications in terms of co-benefits of combining ambitious local adaptation and global greenhouse-gas emission mitigation, as well as the associated residual risks, and concludes by discussing four important challenges for future research.

Box 1: Coastal settlement archetypes This study uses real-world local cases to analyse SLR risk to four coastal settlement archetypes: Urban Atoll Islands (three cases in Tuvalu, Kiribati and the Maldives); Arctic Communities remote from regions of rapid glacial-isostatic adjustment (GIA) (five cases in northern Russia, western Alaska, USA, and northern Canada); Large Tropical Agricultural Deltas (two cases in the Mekong and Ganges–Brahmaputra–Meghna deltas); and Resource-Rich Cities (three cases in the USA, the Netherlands and China) (Table 1). As these coastal archetypes refer to both large and small populations, are located at various latitudes (polar, temperate and tropical regions), and belong to both the Global North and the Global South, taken together, they are illustrative of a wide range of coastal settlements around the world.

The real-world cases have been selected based on both similarities (e.g. for the deltas: large + tropical + dominated by agriculture), differences (a range of socioeconomic, demographic, and governance characteristics), and the fact that they are relatively well documented in the peer-reviewed literature. The analysis does not develop a specific risk assessment for any of these particular cases, but rather uses them to illustrate a diversity of situations within generic categories of coastal settlements. Each coastal archetype therefore represents an average situation based on a set of specific cases.

This approach reflects our intention to characterize coastal archetypes in order to inform a generic understanding that national and sub-national stakeholders might wish to use for guidance in the process of scoping adaptation before localizing planning and implementation to the vast number of particular locations. Such an approach is relevant for two reasons. First, no standardized local-scale projections combining sea level changes, socioeconomic trends and coastal impacts systematically exist at all specific locations, therefore preventing any systematic comparative analysis. Second, national and sub-national stakeholders as well as regional to international organizations increasingly call for lessons to be learned from best/bad practices on planning and managing coastal risks, making comparative case study analyses of critical importance today (see Box 2).

Box 2: Advances This risk assessment has been developed in the conceptual frame of the IPCC Reasons for Concern (RFC), which describes potentially dangerous anthropogenic interference with the climate system^15^. Four major areas of scientific advances are the assessment method, the geographical scale of analysis, the consideration of adaptation scenarios, and the targeted audience.

First, to overcome the lack of consistent local SLR and socioeconomic projections across all the case studies considered (Box 1), and deliver an innovative analysis of the potential benefits of additional adaptation in terms of SLR risk reduction, we develop a formal expert judgment approach (see “Methods”) to describe climate risk in a consistent way across coastal settlement archetypes. We argue that, in the absence of robust and comparable scientific data on the multiple dimensions of risk (hazard, exposure and vulnerability, and adaptive capacity) across a wide diversity of particular locations, assessments based on formal expert judgment using coastal settlement archetypes informed by real-world and well documented cases are of high value to describe climate risks. Of course, uncertainty is associated with such a method, given that different experts could come up with different outcomes (here, scores); this bias is inherent to expert judgment exercises and a way to limit it is to develop clear justifications for each score (see “Methods”) as well as a multiple-round approach.

Second, while earlier RFC-related risk assessments focused on the global level or, at best, very broad regions^22,23^, here we adopt a regional-to-local perspective in order to better understand the spatial variability of risk. A set of real-world coastal cases illustrates risk to human assets (including populations, infrastructure and livelihoods) in four coastal settlement archetypes (Box 1). In that way, our approach injects the regional-to-local perspective into global analyses of risk and adaptation potential, which in the past have been unable to capture on-the-ground realities adequately, or to integrate context-specific considerations of adaptation. On the other hand, most adaptation literature is very focused on specific case studies and fails to make broader generalizations^18^.

Third, we consider two contrasting adaptation scenarios (see “Methods”) covering the range of responses that may be required. The ‘None-to-moderate adaptation’ scenario assumes no major additional adaptation efforts compared to today (e.g., moderate raising of existing coastal protection structures). The ‘High adaptation’ scenario refers to an ambitious and effective combination of both incremental and transformational adaptation (e.g., upgrading coastal protection and relocation of entire settlements, respectively), assuming minimal financial, social and political barriers to the implementation of adaptation measures.

Finally, refining the geographical scope together with including potential benefits for risk reduction allows the range of the users of global-scale papers and reports to be broadened. For example, the original audience of the IPCC reports is two-fold: the international climate negotiation arena, including both working groups under the United Nations Framework Convention on Climate Change (UNFCCC; e.g. the Adaptation Committee and the Subsidiary Body for Scientific and Technological Advice) and national delegations (and subsequently national-level decision-makers); and the global community at large, i.e. a black box including all layers of societies (NGOs, media, general public, etc.). The downscaled perspective in this paper helps address more specific decision-makers’ concerns, for instance those of low-lying coastal continental or island territories experiencing high population and asset densities. This represents a critical step forward as adaptation concerns are growing at the local scale worldwide. At the same time, non-state and sub-national stakeholders are increasingly recognized as important influencers of global processes such as the international climate negotiations (e.g. the C40 network gathering the world’s megacities committed to tackling climate change and its impacts).

## Threats from sea level rise

The sections below present the range of impacts, drivers of risk and sea level scenarios that we used a bases to develop the risk assessment framework.

### Range of impacts

**Figure 2:**
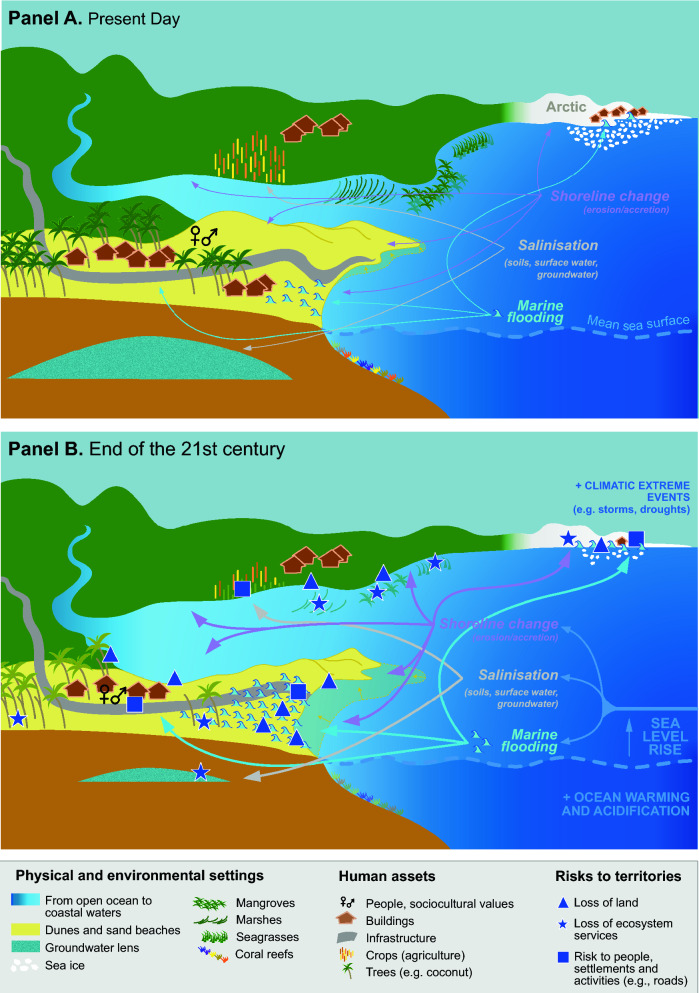
Schematic visualisation of SLR impacts by the end of the twenty-first century. The diagram represents a hypothetical coastal area composed of both tropical, temperate and polar coasts, in the face of marine hazards (flooding, salinisation, and shoreline change) and impacts, including the effects of SLR, for the present-day (**A**) and by the end of this century (**B**). (**A**) That coastal impacts are already occurring, especially shoreline retreat (e.g. shaded sandy shores and yellow arrows), flooding (blue waves symbols) and ecosystem degradation (shaded coastal vegetation and coral reefs). Future impacts are represented with coloured arrows and dark blue triangles, stars and squares (loss of land, loss of ecosystem services, and risk to human assets, respectively). The thickening of the arrows from (**A**) to (**B**) illustrates the increasing influence of SLR. The comparison between (**A**) and (**B**) shows SLR risks in terms of the reconfiguration of the coast (mainly coastal recession here) in all environments; the reduction in size and salinisation of groundwater lenses; the salinisation of soils used for coconut trees and crops (shaded trees and yellow and brown icons); the degradation of coastal vegetation and coral reefs (shaded green and coral icons) partly also due to ocean warming and acidification; and the loss of human assets (e.g. houses and roads). Finally, it shows the expected decrease in sea ice that will amplify SLR effects through reduced physical protection of the land from wave action.

Sea level rise threatens coastal zones through a range of hazards and impacts^17^ including enhancement of episodic, temporary marine flooding due to the effect of rising mean sea level and extreme sea levels associated with storm surge and high tides; permanent submergence of land; groundwater inundation when porous substrates exist; erosion of land and shorelines; change and loss of coastal ecosystems; salinization of ground and surface waters, and impeded drainage of natural or human hydrological systems (Fig. 2).

The loss of land and ecosystem services is of particular concern worldwide. It remains difficult to quantify land loss because of key uncertainties with respect to local ecosystem and shoreline responses to rising seas, as illustrated for instance by the scientific debate on whether atoll islands will disappear or resist shrinkage^24–26^, as well as the extent and effectiveness with which humans will protect shorelines^27^. In addition, key ecosystem services may be lost in particular if coastal defences and other human infrastructure hamper coastal ecosystems’ natural adjustment capacity to SLR, e.g. through inland migration^7,17^. Of particular importance is the loss of coastal protection services^28–30^, as mangroves, corals, saltmarshes and seagrass meadows currently protect hundreds of millions of people worldwide against storm surges and waves^31,32^. For example, under RCP8.5 by 2100, a 1 m loss in coral reef height would more than double the global area flooded during a 100-year event^29^.

Flooding, erosion and salinization pose a wide array of risks to people, settlements and activities including agriculture, tourism, fisheries, and aquaculture^17^. For example, expected global annual flood damages to buildings are projected to increase by 2–3 orders of magnitude if no adaptation measures are implemented^18,27^. These risks are further exacerbated by other climate drivers unrelated to SLR, e.g. permafrost thaw and sea ice retreat in the Arctic^4,33^.

### Non-climatic anthropogenic drivers of risk

Settlement trends fueled by population growth and demographic changes, urbanization and rural exodus, displacement of some indigenous communities, etc. have played a role over the twentieth century in changing coastal populations’ exposure and vulnerability worldwide^34–36^ in four main ways. First, they have resulted in increased population densities at the coast^2,37,38^ and, together with inadequate building codes and land use planning, caused significant infrastructure and assets to be located in risk-prone areas. For example, ~ 57% of the built infrastructure in Pacific Island countries are located in risk-prone coastal areas^39^, and 6–8% of the population living in Latin America and the Caribbean are at high risk of being affected by coastal hazards^40,41^. Second, settlement trends resulted in detrimental impacts on coastal natural environments and resources, and consequently on ecosystem services such as coastal protection^30^ and healthy conditions for fisheries and aquaculture. Locally, anthropogenic drivers include, among others, land reclamation and sand mining for construction, coastal vegetation clearing (e.g., mangroves), reduction of coastal accommodation space for ecosystem adjustment (particularly landward migration of wetlands) and loss of traditional ecological knowledge^17^. Third, settlement trends contributed to human-induced subsidence, especially in deltas and megacities, through land use changes (including asset densification), upstream sediment starvation, and groundwater pumping^42^. Fourth, on a more positive note, coastal societies in many cities and highly exposed areas have reduced coastal risks through improved coastal defences and accommodation (e.g. early warning systems and cyclone shelters, raising land and islands) even in regions that have experienced a twentieth century relative SLR of a meter or more^17^.

The two main conclusions to be drawn from this are, first, that the important contribution of non-climatic anthropogenic drivers to risk makes it difficult to attribute observed impacts only to climate change-induced SLR^17^. And, second, that in the absence of major additional adaptation efforts compared to today, human factors will continue to increase exposure and vulnerability in many locations, concomitant with the increasing influence of SLR.

### Projections of SLR

Global mean sea level (GMSL)^17,43^ will have risen by 0.43 m (0.29–0.59 m, likely range; RCP2.6) and 0.84 m (0.61–1.10 m, likely range; RCP8.5) in 2100, relative to 1986–2005. The corresponding end-century rates of GMSL rise^17^ are between 4–9 mm/year and 10–20 mm/year (RCP2.6 and RCP8.5 likely ranges, respectively) compared to 3–4 mm/year since the early 1990s. Building on AR5^44^ and associated CMIP5 simulations, these projections include the outcomes of several recent studies^45,46^ that better quantified the dynamic contribution of the Antarctic ice sheet. These scenarios consider the importance of combined hydrofracturing and marine ice cliff instability to be negligible over this century due to the existence of ice shelves for which a widespread melt is not foreseen by high resolution regional climate models^17^. Recent developments explored a low-likelihood, high-impact scenario including ice loss from Antarctic Ice Sheet, which could contribute to more than 1 additional meter of sea level rise by the end of this century^43^. However, given the lack of impact literature associated with this extreme scenario, we did not considered it.

For risk assessment pertinent to specific locations (Box 1), sea level projections are needed at regional or higher resolution. There is, however, no universal, agreed-upon modelling approach that uses consistent methodologies for local-scale SLR projections (and associated coastal hazards) across a wide diversity of locations and coastal configurations, while such a consistency is critical to any precise comparative approach. Although it is possible to project site-specific sea level extremes (based for example on a statistical analysis of tide gauge records)^47,48^, several knowledge gaps prevent us from going beyond regional-level mean SLR projections for specific coastal locations. First, future changes in the rates of human-induced subsidence, which play an important role locally^49^, were not considered due to the unpredictable future of human behavior controlling the current trends^50^. Here we hypothesize the continuation of the latter trends. Second, while mean SLR will push extreme sea levels upward, there is insufficient agreement on how changes in extreme event climatology will alter extreme sea levels at specific coasts^17^. Here we therefore consider the part of changes in extreme sea levels due to SLR only, therefore excluding changes in the climatology of the storms that cause those changes. Third, it is increasingly recognized that changes in local shorelines (and, of course, sea ice) over this century will strongly affect the local wave climate and its effect on extreme sea levels, but such forward-looking studies remain in their infancy^28,51^. Accordingly, this paper uses mean regional SLR projections for 2100 and their effect on regional extreme sea levels as a starting point for assessing risk at the coastal settlement archetype level (see “Methods”).

Following the most common method, we combine local extreme sea level statistics from tide gauge records with projections of regional mean sea level (RSL) rise. RSL is derived from model projections^17^ at ~ 100 km resolution. Regional projections include gravitational and rotational effects due to changes in ice mass and land water storage, regional differences in the steric component and ocean dynamics, and glacial-isostatic adjustment (GIA). They do not include human-induced subsidence patterns. This study assesses risk for the median of RCP2.6 and RCP8.5, and the upper likely range of RCP8.5 (Table 1﻿).

**Table 1:** Global mean and regional sea level rise (GMSL, RSL) by 2100 at the global scale and for the case studies used for the risk assessment. In the Arctic, only coastal communities (*) remote from regions of rapid glacial isostatic adjustment (GIA) have been considered. Anthropogenic subsidence is not included in these projections. Source: Ref.^17^.

Low-lying coastal archetypes	Location	Sea level change (in m)			
		RCP2.6	RCP8.5		GIA
		Median	Median	Upper range (> 95%)	
**Global Sea Level Mean (GMSL)**		0.43	0.84	1.10	–
Urban atoll islands	South Tarawa Urban District (Kiribati)	0.49	0.92	1.32	− 0.02
	Fongafale (Tuvalu)	0.49	0.91	1.33	− 0.01
	Male (Maldives)	0.46	0.92	1.32	− 0.01
	**Mean**	**0.48**	**0.92**	**1.32**	**–**
Arctic coastal communities*	Bykovskiy (Russia)	0.34	0.79	1.17	− 0.01
	Shismaref (Alaska, USA)	0.40	0.81	1.13	0.07
	Kivalina (Alaska, USA)	0.37	0.77	1.10	0.06
	Tuktoyaktuk (Canada)	0.39	0.77	1.09	0.18
	Shingle Point (Canada)	0.40	0.76	1.10	0.17
	**Mean**	**0.38**	**0.78**	**1.12**	**–**
Large tropical agricultural deltas	Mekong (Vietnamese portion)	0.43	0.84	1.23	− 0.04
	Ganges–Brahmaputra–Meghna (Bangladeshi portion)	0.33	0.74	1.08	− 0.04
	**Mean**	**0.38**	**0.79**	**1.16**	**–**
Resource rich cities	New York City (USA)	0.55	1.02	1.53	0.09
	Rotterdam (The Netherlands)	0.39	0.82	1.23	0
	Shanghai (China)	0.42	0.84	1.29	− 0.03
	**Mean**	**0.45**	**0.89**	**1.35**	**/**

## Risk levels and adaptation benefits

**Figure 3:**
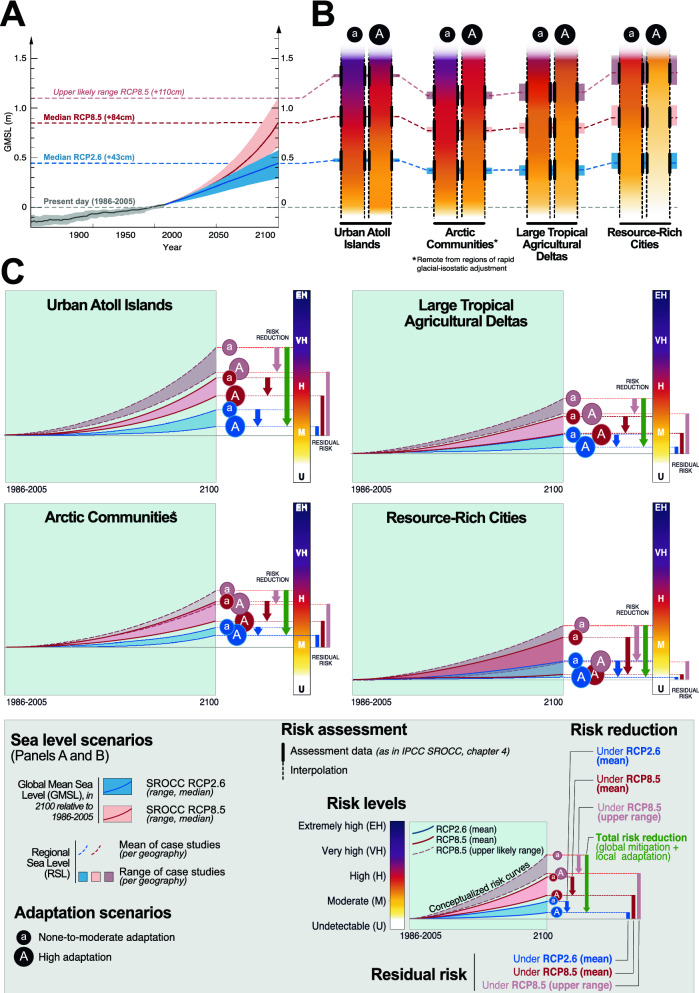
Additional SLR risk to low-lying coasts and adaptation benefits over the twenty-first century. In Panel A, GMSL serves as a generic descriptor of climate change scenarios, while the risk assessment is based on end-century regional sea level rise (RSL; background SLR information on Panel B). RSL is composed of several regionally differentiated contributions (see “Projections of SLR” section and “Methods”) for each of the 13 real-world case studies used to describe the four coastal settlement archetypes (see Table 1), and mean and upper likely range values of RSL per coastal archetype are used for the risk assessment. Human-induced subsidence is not included in the RSL projections: although acknowledged to be important at several locations, especially deltas and megacities, human-induced subsidence is too difficult to project to the end of the century within reasonable uncertainty—N.B.: the assessment however does take account of abatement measures implemented in response to current rates of subsidence in scoring risk and risk reduction. Panel B shows SLR risk for the settlement archetypes today and in 2100, under RCP2.6 and RCP8.5 and under two adaptation scenarios (“None-to-moderate” vs. “High” adaptation; see “Methods” for description). Risk assessment has been conducted for each SLR and adaptation scenario, while intermediate risk levels are interpolated (see the solid and dotted burning embers’ outlines). Panel C builds on Panel B to illustrate the SLR risk reduction through local adaptation (blue, red and light brown vertical arrows for RCP2.6 median, RCP8.5 median and RCP8.5 upper likely range, respectively) and in combination with global mitigation (green arrows). It also illustrates residual risks for each SLR scenario (blue, red and light brown vertical bars). The positioning of end-century risk levels for settlement archetype precisely reflects the SROCC assessment scores (see SM2). Risk development curves are hypothetical and based on SLR projection curves (**A**).

This section discusses the end-century risk assessments for each coastal settlement archetype (Fig. 3B), and the potential benefits of a range of adaptation responses (Fig. 3C, “Methods” and Table 3). The risk language is inspired by the IPCC^4^ and distinguishes between four main qualitative levels, from *Undetectable* (risks are undetected) to *Moderate* (detectable with at least medium confidence), *High* (significant and widespread), and *Very high* (very high probability of severe risks and significant irreversibility or persistence of impacts). For the purpose of this study—new compared to the SROCCframing—we added a fifth level describing *Extremely high* risk as a very high probability of severe and irreversible risks exceeding the coping capacity of the affected socioecological systems; and, therefore threatening the habitability of human settlements^52,53^ and possibly leading to existential or catastrophic risk^54,55^.

Quantitative estimates complement the IPCC qualitative descriptors. They are based on a scoring system developed by the authors to assess risk amplification/reduction across SLR and adaptation scenarios (“Methods”) and, new compared to the SROCC analysis, are presented as relative percentages (integer) along the *Undetectable*-to-*Extremely high* risk scale (Supplementary Material SM1). The quantitative estimates do not suggest that SLR risk levels can be quantified on an absolute scale, but are rather used for an illustrative purpose in order to complement the IPCC qualitative risk language and allow for increasing the consistency of interpretation among a wide diversity of users^14,56^. In addition to systematically associate qualitative and quantitative statements in the aim of limiting interpretation biases, we assigned equal weight to individual criteria scores in order to avoid any value-judgment on the relative importance of the risk drivers. The final results are synthesized in Table 2 (SM2 for detailed version) and Fig. 3C, and described in the sub-sections below for each coastal archetype and following the same structure: current situation, key controlling factors of future SLR risk (as considered in this study) and associated risk level estimates, and role of adaptation in reducing risk levels.

**Table 2:** Main results. See “Methods” section for explanations and SM2 excel file for detailed results. Columns M1-M9 and (1) represent the main results from the SROCC analysis, i.e. individual and aggregated scores per SLR scenario (a + 45 cm, + 83 cm, + 110 cm) and adaptation scenario (None-to-moderate (A), High (B)). Columns (2) and (3) present new analysis compared to the SROCC and form the bases of this paper. They describe the integer percentages used in the main manuscript in terms of the aggregated risk scores along the *Undetectable*-*Extremely high* risk scale (2) and the associated level of risk reduction per SLR scenario (3). Source: Ref.^17^.

Scenarios		Assessment metrics (drivers of risk and adaptation)									Aggregated results		
GMSL	Adaptation	M1	M2	M3	M4	M5	M6	M7	M8	M9	(1)	(2) in %	(3) in %
**Resource-rich cities**													
Present-day		6	1	2	1	0	− 3	0	− 1	0	6	8.0	–
+ 43 cm	(A)	7	2	4	2	0	− 1	0	− 1	0	13	17.3	− 9.3
	(B)	6	1	1	1	0	− 3	0	0	0	6	8.0	
+ 85 cm	(A)	10	4	7	3	0	0	0	− 2	0	22	29.3	− 20.0
	(B)	6	1	2	1	0	− 3	0	0	0	7	9.3	
+ 110 cm	(A)	12	5	10	3	0	0	0	− 3	0	27	36.0	− 20.0
	(B)	8	2	4	1	0	− 2	0	− 1	0	12	16.0	
**Urban Atoll Islands**													
Present-day		5	5	5	4	2	− 2	− 1	0	0	18	24.0	–
+ 43 cm	(A)	7	7	8	6	4	− 2	− 1	0	0	29	38.7	− 9.3
	(B)	7	7	8	6	4	− 4	− 3	− 3	0	22	29.3	
+ 85 cm	(A)	10	9	11	8	6	− 2	− 1	0	0	41	54.7	− 9.3
	(B)	10	9	11	8	6	− 4	0	− 6	0	34	45.3	
+ 110 cm	(A)	13	11	14	10	8	− 2	− 1	0	0	53	70.7	− 13.3
	(B)	13	11	14	10	8	− 4	0	− 9	0	43	57.3	
**Large tropical agricultural deltas**													
Present-day		4	3	3	2	2	− 2	− 1	0	0	12	16.0	–
+ 43 cm	(A)	4	4	5	3	4	− 2	− 1	0	0	18	24.0	− 8.0
	(B)	4	4	5	3	4	− 3	− 3	0	− 2	12	16.0	
+ 85 cm	(A)	4	5	8	5	6	− 2	− 1	0	0	26	34.7	− 10.7
	(B)	4	5	8	5	6	− 4	− 2	− 3	− 1	18	24.0	
+ 110 cm	(A)	4	5	11	7	8	− 2	− 1	0	0	33	44.0	− 8.0
	(B)	4	5	11	7	8	− 5	− 1	− 3	0	27	36.0	
**Arctic communities**													
Present-day		4	5	4	5	2	− 1	0	− 1	0	18	24.0	–
+ 43 cm	(A)	5	7	6	8	2	− 1	0	− 1	0	26	34.7	− 4.0
	(B)	5	7	6	7	2	− 2	0	− 2	0	23	30.7	
+ 85 cm	(A)	6	10	8	11	3	− 1	0	− 1	0	36	48.0	− 8.0
	(B)	6	10	8	10	3	− 3	0	− 4	0	30	40.0	
+ 110 cm	(A)	7	11	9	12	3	− 1	0	− 1	0	40	53.3	− 6.7
	(B)	7	11	9	12	3	− 3	0	− 4	0	35	46.7	

Assessment metrics: see Table 3 for detailed description of M1 to M9.

Aggregated results: (1) = aggregated risk score; (2) = % against end-century score range and along the *Undetectable*-*Extremely high* risk scale (0–75); (3) level of risk reduction per SLR scenario (in %, note that integer percentage are used in the main text).

**Table 3:** The indicators used in this risk assessment. See “Methods” section for explanations.

Risk dimension	Indicator	Brief description
**Exposure and vulnerability**	M1 **Density** of assets (population, buildings, infrastructure)	High densities of population and built assets contribute to high exposure to coastal hazards, especially under conditions of relative land scarcity (e.g. in atoll islands or when constraining land uses). In most cases, the types of buildings, location of critical infrastructures, socioeconomic segregation, etc. lay the foundation for vulnerability Scenario considered for the twenty-first century: relatively stable density levels over the century (one plausible scenario among many). The potential for decrease in assets density is considered through M8
	M2 Level of degradation of **marine and terrestrial natural buffers**	Ecosystems provide coastal protection services to human communities, as illustrated by coral reefs and the associated beach-dune systems, for example, through wave energy attenuation (i.e. wave breaking over the reef crest and wave friction over the reef flat, reduction of wave run-up due to the absorption and dissipation of the remaining wave energy by the coastal sedimentary system), and carbonate sediment supply to the coast. Natural buffers considered in this study are marine (coral reefs, mangroves, wetlands and sea ice) and terrestrial (beaches, dune systems and vegetation) Scenario considered for the twenty-first century: continued degradation at the same pace than recent trends. Response to this scenario is captured in M7
**Hazard**	M3 Relative extend of **direct marine flooding**	Direct marine flooding results from the effect of rising mean sea level on extreme water levels associated with storm surge and high tides. Direct marine flooding can be temporary in case of extreme events, or lead to permanent submergence. One major secondary impact of marine flooding is the salinisation of affected areas (see M5). Note that in this assessment, we underestimate the role of groundwater inundation as a result of porous substrates
	M4 Degree of **coastal erosion** (beaches and/or dune systems) or permafrost thaw	Coastal erosion refers to shoreline retreat and the progressive loss of land. Permafrost thaw is fuelled by air and ocean warming and SLR, and results in shoreline retreat
	M5 Degree of **salinisation** of groundwater lenses, soils and surface waters	Saline water intrusion into coastal aquifers and surface waters and soils is exacerbated by rising sea levels, drought events and decreasing river discharges in combination with human-induced water extraction. While SLR is only one of the two main controlling natural factors of aquifers volume and quality –the other is precipitation–, even a small rise in sea level can have substantial effects on aquifers, especially in atoll island contexts. Overall, salinisation especially impacts coastal agriculture and freshwater availability
**Adaptation**	M6 Implementation level of adequately calibrated **hard engineered coastal defences**	M6 refers to “grey” or “hard” coastal protection structures, especially dykes, seawalls, rip-raps and groynes. They provide quite predictable levels of safety, but require technical maintenance (and funding) over long time periods (several decades) to remain efficient. This raises the affordability issue that explains why hard coastal protection is usually considered a long-term option in densely populated areas, but not in rural and poor areas. Hard coastal protection is also recognized to result in the loss of natural coastal dynamics that can play an important role in shoreline stability, especially in rural areas
	M7 Implementation level of **restoration of degraded ecosystems**, or creation of new natural buffers areas	This indicator is complementary to M2 and is used as a proxy for ecosystem-based adaptation, a strategy that is gaining traction worldwide
	M8 Implementation level of **planned and local-scale relocation** of people, assets and activities	The relocation of people and assets locally, i.e. inland or in nearby neighbouring areas, can offer a solution before considering definitive displacement of people and activities nationally or even internationally. This question however raises cultural, ethical (i.e. who should leave?), economic (e.g., loss of jobs locally, competitiveness issues in the destination area) and political (i.e. difficult governance arrangements) issues. These issues have been debated in the context of the approval session of the SROCC (with national delegations; Sept. 2019), and led to some refinements, especially on two points: (1)This assessment takes into consideration the specific physical constraints of each coastal settlement archetype. In particular, while megacities and deltas have a hinterland for relocation within the territorial system, land scarcity in atoll islands implies that relocation can take place within the island if relocation needs are moderate, but must be either in another neighbouring island or in artificially raised islands in the case of higher relocation levels (but still at a local scale); (2)M8 refers to planned relocation aiming at reducing the exposure of people, assets and infrastructure, and not to spontaneous relocation by individuals or small communities. In addition, M8 refers to proactive managed retreat or resettlement only at a local scale, and according to the specificities of a particular context. Resettlement at a larger scale is excluded; as well as forced displacement and international migration are not considered adaptation in the context of this study and, as a consequence, are also excluded from M8
	M9 Limit **human-induced subsidence**	Subsidence refers to downward motion of the land surface and therefore has a strong influence on relative sea levels and sea-level rise. M9 considers measures addressing anthropogenic subsidence resulting from local extractive activities as well as major human disturbances to sediment supply, for example, due to fresh water exploitation or damming and land use change upstream from the coast

One additional important clarification touches on one of the four adaptation responses considered in this assessment (“Methods”, Table 3), i.e. planned and local-scale relocation of people, assets and activities inland or in nearby neighbouring areas. The relocation issue raises major cultural (e.g. loss of identity), ethical (e.g. right to stay, who should leave?), economic (e.g. loss of jobs locally, competitiveness issues in the destination area) and political (i.e. difficult governance arrangements) concerns involving different world views. In this study we do not consider relocation as climate adaptation a priori, but argue that planned relocation, unlike spontaneous and forced relocation, could contribute to reduce the exposure and vulnerability of people, assets and infrastructure over generations. International migration is not considered within the relocation response.

### Urban Atoll Islands

The assessment builds on the examples of the capital islands of Fongafale (Tuvalu), Male’ (Maldives) and South Tarawa (Kiribati). All are very low-lying (< 4 m above mean sea level, with lagoon coasts < 2.00 m in South Tarawa)^57,58^ and composed predominantly of reef-derived unconsolidated material. They are home to a high proportion of these countries’ inhabitants (63.1%, 32.0% and 49.3% for Tuvalu, the Maldives and Kiribati, respectively), economic activities and critical infrastructure. The coastal protection services delivered by ecosystems, including wave energy attenuation and sediment provision and trapping^28,59^, have been increasingly undermined by local human disturbances over the past decades^24,30^, e.g. through pollution, land reclamation^59^ and sediment mining^58^. The current SLR risk level is estimated *Moderate* (24% on the *Undetectable*-*Extremely high* risk scale).

Key controlling factors of future SLR risk are trends in marine flooding and coastal erosion, human asset density and decreasing coastal protection ecosystem services. Flood events have increasingly affected these islands^60,61^, and more combinations of mechanisms including wave-driven direct flooding (surface) and groundwater inundation (as a result of porous substrates) are to be expected over the coming decades^62^. Coastal erosion affects the unprotected shorelines of Fongafale^63^ and South Tarawa^58^, while Male’s shoreline is fixed by hard engineering^64^. High population densities (e.g., ~ 3200 inhabitants/km^2^ in South Tarawa^65^; ~ 65,700 inhabitants/km^2^ in Male’^66^) and critical infrastructure concentration in flood-prone areas^58,61,62^ significantly contribute to risks. As a result, and given that we underestimate here the role of groundwater inundation (see above), we estimate a relatively moderate increase of SLR risk by 2100 under a 0.43 m GMSL rise (0.43–0.49 m range for RSL in the three locations considered; Table 1), from *Moderate* today to *Moderate-to-high* (i.e. from 24 to 39% on the *Undetectable*-*Extremely high* risk scale). Under RCP8.5 median and upper likely range (0.84–0.92 m and 1.10–1.33 m respective RSL ranges), end-century risk will respectively increase to *High* and *Very high* levels (55 and 71% on the *Undetectable*-*Extremely high* risk scale). In Tuvalu, for example, the proportion of population living below annual flood levels, currently 59%, will increase by 10.2% percentage points in the case of 1 m SLR by 2100, in the absence of effective adaptation^12^.

High adaptation is expected to reduce end-century SLR risk from *Moderate-to-high* to slightly below this level under RCP2.6 (from 39 to 29% on the *Undetectable*-*Extremely high* risk scale), and from above to below *High* under RCP8.5 median (from 55 to 45%). The two main adaptation options considered in this assessment are adequately calibrated hard-engineered coastal defences and planned local relocation, as we assume that already degraded ecosystems will increasingly be unable to cope with SLR^11,67–69^. Male’ is the only capital island in this study to be entirely surrounded by a double line of engineered coastal protection structures (mainly breakwaters and seawalls^64^), while South Tarawa^57^ and Fongafale^61,63^ are only partially protected, for the most part with artisanal vernacular structures. Due to the permeable nature of the island substrate, even adequate structures might not prevent seawater ingress from below^50^, therefore potentially limiting adaptation benefits as sea level rises. Regarding relocation measures, which remain sporadic and unplanned in most Urban Atoll Islands, the assessment assumes that proactive relocation on the same island or to a nearby island (e.g. to Hulhumale’ for Male’)^70^ could help offset the extent of flooding-related impacts under RCP2.6. It also assumes that as sea level rises, enhanced planned local relocation will continue to help limiting risk even under RCP8.5 upper likely range, and despite increasing constraints due to land scarcity, economic challenges and social reluctance^50,71^. Adaptation benefits under RCP8.5 upper likely range are therefore estimated higher than under RCP8.5 median, lowering risk level from close to *Very high* to slightly above *High* risk (i.e. from 71 to 55%).

### Arctic communities

While some Arctic communities lie within regions of postglacial isostatic uplift and falling relative sea level, even for RCP8.5 projections^72^, our assessment builds on the examples of five small settlements with slow to moderate SLR on the Arctic Coastal Plain. All have seasonal sea ice along coasts formed in unlithified but ice-rich sediments in permafrost. Bykovsky (Lena Delta, Sakha Republic) is mainly situated on an ice-rich, eroding terrace ~ 20 m above sea level^73^; Shishmaref and Kivalina (western Alaska) are located on low-lying barrier islands susceptible to rising sea level and storms^51,74^; Shingle Point and Tuktoyaktuk (Mackenzie Delta, Canada) are respectively on a gravel spit and low tundra with extensive massive ground ice^72^. This archetype is characterized by small populations (< 900 inhabitants), mostly Indigenous, heavily dependent on marine subsistence resources and transportation by sea or on ice^72^. Anthropogenic drivers include a history of (re)settlement in marginalised, climate-sensitive communities at vulnerable coastal sites. As a result, current risk level is estimated as *Moderate* (24% on the *Undetectable*-*Extremely high* risk scale).

Key components of future risk include accelerated coastal erosion and reduced sea ice (more open water, thus increased wave exposure) at all sites^51,72,75^, exacerbated by SLR. Permafrost thaw and loss of ground-ice volume is a particular issue at Bykovsky and Tuktoyaktuk^73,75^ but contributes to widespread acceleration of coastal erosion in the circumpolar Arctic. All sites but Bykovsky are currently at risk of more frequent storm-surge flooding, threatening infrastructure, cultural sites, health and safety. Winds causing upwelling prior to storm flooding may facilitate salinization in the outer Mackenzie Delta^10,76^, where SLR combined with natural subsidence threatens loss of globally important nesting habitat through sustained inundation^10^. Figure 3C shows that, without enhanced adaptation and as for Urban Atoll Islands, these sites collectively face *Moderate-to-high* risk by 2100 even under RCP2.6 median (0.34–0.40 m RSL range in Table 1; 35% on the *Undetectable*-*Extremely high* risk scale). Under progressively higher SLR, the risk is assessed as *High* for RCP8.5 median (48% on the *Undetectable*-*Extremely high* risk scale), and above *High* for RCP8.5 upper likely range (1.09–1.17 m RSL range; 54% on the *Undetectable*-*Extremely high* risk scale).

High adaptation is expected to have relatively limited benefits (Fig. 3C). Some options, such as hard coastal defences and the restoration of degraded ecosystems, are confounded by additional factors, including both diminishing sea ice protection and rising temperatures promoting rapid erosion and thermokarst destabilization^74^. Planned and local-scale relocation is constrained by the limited suitable land base. More substantial relocation has been considered but not yet implemented at Kivalina and Shishmaref due especially to institutional barriers and cost^77^. Considering the range of adaptation options and their viability in the context of additional change independent of SLR, we estimate that high adaptation can only slightly reduce collective risk to above *Moderate*, *Moderate-to-high* and *High* under RCP2.6, RCP8.5 median and RCP8.5 upper likely range, respectively (31, 40 and 47% on the *Undetectable*-*Extremely high* risk scale).

### Large tropical agricultural deltas

Two large, low-lying and mainly agriculture-dominated deltas are considered, the Mekong (focus on the Vietnamese portion) and the Ganges–Brahmaputra–Meghna (GBM; Bangladeshi portion). For each delta portion, we consider the entire delta area (as opposed to the coastal fringe only) in order to capture the interconnected nature of deltaic landscapes and associated risks. Both deltas are prone to riverine, tidal, and storm-surge flooding^78,79^. High tides and cyclones can lead to large and deadly marine flooding events, especially in the GBM delta. Coastal and river bank erosion is also affecting both deltas^80^ and saline intrusion impacts their coastal aquifers, agricultural land, and surface waters^81,82^.

Risk from multiple hazards is driven by exposure of population (~ 1280 and 433 inhabitants/km^2^ in the GBM and the Mekong delta, respectively) and of agricultural land, with agriculture contributing strongly to the GDP of both countries (14.7% and 13.1% in Vietnam and Bangladesh, respectively, in 2018)^83,84^. Human-induced subsidence^85,86^ and the removal of natural vegetation buffers such as mangroves and other wetlands^87,88^ exacerbate coastal risks. Both deltas are partly protected with dykes to prevent riverine flooding, sea walls to prevent marine flooding, and polders and sluices in some coastal stretches to prevent salinity intrusion during storm surges^83,89^. As a result, we estimate that Large Tropical Agricultural Deltas currently face a below *Moderate* SLR risk level (24% on the *Undetectable*-*Extremely high* risk scale). Under a 0.43 m rise in GMSL by 2100 (RCP 2.6; 0.33–0.43 m RSL range; Table 1) and without any substantial additional adaptation efforts, risk level will increase to *Moderate* (35% on the *Undetectable*-*Extremely high* risk scale), and is expected to become close to *High* under a 1.10 m rise in GMSL (RCP8.5 upper likely range; 1.08–1.23 m RSL range; 44% on the *Undetectable*-*Extremely high* risk scale). Even higher risk levels are possible for the Mekong delta, given that a recent study indicates much lower mean elevation that previously thought (i.e., ~ 0.8 m above sea level compared to ~ 2.6 m)^90^. Key components of future risk include a stronger contribution of marine flooding^85,91^, coastal erosion^91–93^ and salinization of coastal waters and soils^94,95^, with substantial consequences for agriculture and water supply^96^. Besides SLR, risk increase will be fueled by the continuation of human-induced subsidence^82,86^, the development of upstream dams and reservoirs affecting water and sediment flows, and other more local factors such as sand mining, that however remain very difficult to forecast at the century scale.

High adaptation is expected to limit SLR risk to below *Moderate* under RCP2.6, *Moderate* under RCP8.5 median, and *Moderate-to-high* under RCP8.5 upper likely range (respectively 16, 24 and 36% on the *Undetectable*-*Extremely high* risk scale). Combinations of measures to be considered include the prevention of further subsidence and enhancement of aquifer recharge; engineered defences such as dykes and sluice gates^97,98^; the development of accommodation measures such as early warning systems^99^; and ecosystem-based approaches to stabilize the shoreline and riverbanks, and enhance buffering ecosystem services^100^. Further strategies include, for example, “living with salinity” through the adaptation of agriculturally-based livelihoods, and in the GBM delta (despite collateral effects in terms of relocation), tidal river management in which land is raised using sediments captured through controlled flooding^101^. Our assessment considers planned relocation out of the most exposed areas as an option only for higher emission scenarios.

### Resource-rich cities

Real-world illustrations considered here are New York City (NYC, USA; 8.6 million inhabitants in 2019), Shanghai (China; 24.3 million inhabitants), and Rotterdam (The Netherlands; 0.6 million inhabitants), all situated where major rivers drain into the ocean. These examples represent a range of responses to date to a high level of coastal hazard in circumstances where resource availability is relatively high but each cities’ governance system is distinct^102–104^. Individually and together, they also encompass a range of geophysical settings but our assessment focuses primarily on risk to the high-density urban sections of each. NYC is located at the mouth of the Hudson River where it meets the North Atlantic. The densest parts of the urban center are underlain by granite, and many low-lying and swampy areas fringing Manhattan have been reclaimed and settled over centuries. Surrounding areas within the City limits have lower human asset density and the lowest lying neighbourhoods border directly on the ocean. Shanghai is located on the Yangzte River delta where the river empties into the East China Sea. The central city is based on compacting sediment and consequently experiences recent subsidence rates of about 5 mm/year (including a contribution from tectonic processes)^102^, about three times NYC’s rate, the latter primarily due to GIA. Rotterdam locates at the mouth of the Nieuwe Maas channel of the Rhine–Meuse–Scheldt delta. Most of the city lies below sea level^105^ and experiences high rates of human-induced subsidence (7–13 mm/year)^106^.

NYC’s economic hub, Manhattan’s financial district, is highly exposed to storm surges. The entire coastline of the city is exposed to occasional tropical cyclones (hurricanes) and frequent extratropical cyclonic storms, both of which can be accompanied by storm surge, so that storm tides frequently overtop protective walls, erode sandy beaches, and penetrate barrier islands at the city’s ocean-facing periphery. A few neighbourhoods experience regular tidal (or nuisance) flooding^107^. Shanghai’s terrain is low (average elevation 4 m) and most of the city would be exposed to flooding during intense typhoons if it were not bounded by extensive protection against marine and river flooding^102^. The main increase in coastal hazards for both NYC and Shanghai in the future arises from surge due to tropical cyclones combined with higher sea level (exacerbated by an uncertain future rate of subsidence in Shanghai)^108^. Rotterdam is highly exposed to storm surges that travel up the Rhine^105^. Extensive damage from storm surge in 1953 led to large financial and institutional investments in flood protection^109^. As a result, current risk level is estimated close to *Undetectable* (8% on the *Undetectable*-*Extremely high* risk scale).

This assessment assumes that hard engineered coastal defences will be the dominant, but not sole adaptation response in Resource-Rich cities, supported by some planned local relocation^110^ and enhancement of nature-based protection^111^. Accordingly, and in part as a product of the recent vintage of Rotterdam coastal defences^50,105^, the current situation of this city serves as a reference point for Resource-Rich Cities’ end-century risk levels under high adaptation. As neither NYC nor Shanghai currently achieve that level of defence, and despite significant recent progress such as in NYC in response to Hurricane Sandy in 2012^112^, these cities’ current situations are used to illustrate the none-to-moderate adaptation scenario. In absence of enhanced adaptation, such Resource-Rich Cities will face close to *Moderate* risk by 2100 under RCP2.6 (0.39–0.55 m RSL range in Table 1; 17% on the *Undetectable*-*Extremely high* risk scale). Under progressively higher SLR, the risk is assessed as slightly above *Moderate* for RCP8.5 median (0.84–1.02 m RSL range; 29% on the *Undetectable*-*Extremely high* risk scale), and *Moderate-to-high* for RCP8.5 upper likely range (1.23–1.53 m RSL range; 36% on the *Undetectable*-*Extremely high* risk scale). For cities whose current level of adaptation resembles NYC or Shanghai, efforts to reduce risk to the current level in Rotterdam would require increased hard protection (e.g., surge barriers) for the central city; a combination of hard protection, nature-based protection (e.g., dune and wetland enhancements), and planned local relocation of the most exposed residential areas; and accommodation measures such as elevating some structures. This would allow keeping end-century risk level below *Moderate* under RCP8.5 upper likely range (16% on the *Undetectable*-*Extremely high* risk scale), and close to today’s *Undetectable-to-moderate* level under lower SLR scenarios (respectively 9 and 8% for RCP8.5 and RCP2.6 medians). If rates of subsidence increase from recent levels at Shanghai and Rotterdam, then this assessment would underestimate risk.

### Synthesis: SLR risk across low-lying coastal settlement archetypes

**Figure 4:**
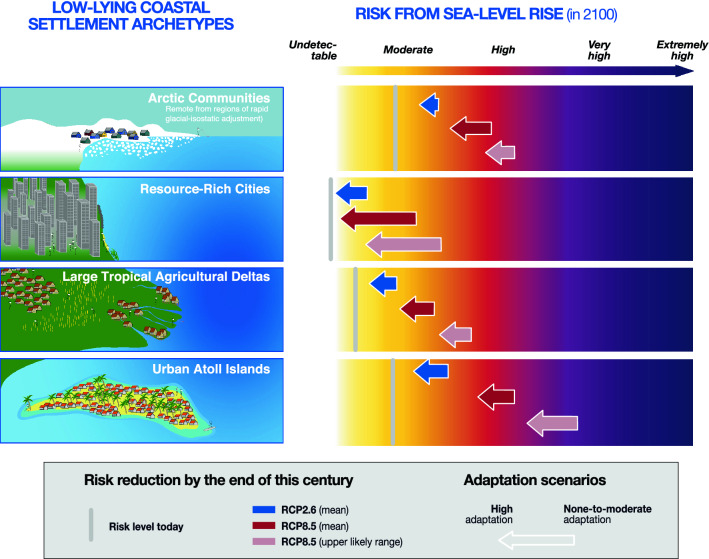
Synthesis on additional SLR risk to a set of low-lying coastal archetypes by the end of the twenty-first century. The left-hand side presents a visualization of the four coastal settlement archetypes analysed in this study. The right-hand side uses the same material as in Fig. 3 (see Table 2 and SM2) to display risk levels under various sea level rise scenarios associated with global warming scenarios, and two adaptation scenarios (none-to-moderate versus high).

This assessment suggests that in the absence of high adaptation and given SLR projections, the additional coastal risks induced by SLR are expected to increase over this century in all low-lying coastal areas, whatever their physical setting (island or continental), location (from Tropics to Poles, except where rapid GIA uplift) and level of development (Fig. 4). Risk escalation will be particularly prominent in Arctic Communities and Urban Atoll Islands, even under RCP2.6: from 24.0% today to respectively 44 and 39% on the *Undetectable*-*Extremely high* risk scale by the end of the century. As SLR will continue, and still considering none-to-moderate additional adaptation efforts compared to today, all settlement archetypes are expected to experience close to *High* to *High-to-very high* risks in 2100 at the RCP8.5 upper likely range (range between 44.0 and 71% on the *Undetectable*-*Extremely high* risk scale), except Resource-Rich Cities where risk will be *Moderate-to-high* (36% on the *Undetectable*-*Extremely high* risk scale). While according to this assessment Urban Atoll Islands and Arctic Communities can be considered at the frontline of SLR risks, it would be misleading to underestimate SLR-related challenges in Large Tropical Agricultural Deltas and Resource-Rich Cities. A key reason is that given the size of the populations concerned (millions of people), the importance of deltas’ agriculture for both local and global food security and trade, and the economic-value of cities’ assets, even a moderate risk level can have substantial consequences.

As an important, innovative step, this assessment demonstrates that implementing ambitious adaptation will be substantially beneficial to the continued habitability of most low-lying coastal settlements over this century, at least until the RCP8.5 median SLR level is reached. Under RCP2.6, all coastal archetypes will benefit from some degree of SLR risk reduction from high versus none-to-moderate additional adaptation (blue arrows in Figs. 3C and 4), especially Urban Atoll Islands and Resource-Rich Cities (risk reduction by 9% compared to risk under no-to-moderate adaptation), and to a lesser extent Large Tropical Agricultural Deltas (− 8%) and Arctic Communities (-4%). Adaptation benefits are expected to substantially increase under RCP8.5 median (red arrows) in Resource-Rich Cities, Large Tropical Agricultural Deltas and Arctic Communities (respective additional risk reductions by 20, 11 and 8% compared to 9, 8 and 4% under mean RCP2.6); and to remain equivalent to RCP2.6-related benefits in Urban Atoll Islands (− 9%). The results however also suggest that as GMSL rises from + 0.83 to + 1.10 m (RCP8.5 upper likely range, light brown arrows), the benefits of in situ additional adaptation, i.e. excluding non-local relocation (see M8 in Table 3), will start to decrease in Large Tropical Agricultural Deltas and Arctic Communities (i.e. smaller arrows; respective risk reduction by 8 and 7% compared to 11 and 8% under mean RCP2.6), and cease to increase in Resource-Rich Cities (i.e. stable arrow height; − 20%). This suggests a possible decrease in in situ adaptation effectiveness at highest levels of warming, therefore confirming a conclusion by O’Neill et al.^22^ that limits to coastal adaptation could occur around a 1.0 m rise in global mean sea level.

## Adaptation potential and limitations

### Coupling mitigation and adaptation

Figure 3 (green arrows in panel C) emphasizes that the end-century level of avoided SLR risk depends on the combination of global mitigation efforts and high local adaptation, and is archetype-specific. First, the end-century additional SLR risk level across all coastal settlement archetypes is considerably higher without ambitious mitigation (RCP8.5 compared to RCP2.6). Second, in all coastal archetypes except Resource-Rich Cities, high adaptation under a high emission scenario (RCP8.5) cannot reduce risks down to a level that would be achievable in a low emission scenario (RCP2.6) without any substantial additional adaptation efforts compared to today. In other words, the failure to effectively mitigate climate change globally cannot be entirely offset through local adaptation, although the latter has the potential to substantially reduce risk in all SLR scenarios.

The full potential of both global mitigation and local adaptation therefore needs to be utilized if SLR risks are to be kept close to today’s level in Resource-Rich Cities, and slightly higher than today in Large Tropical Agricultural Deltas, Urban Atolls Islands and Arctic Communities.

### Unavoidable risks

The assessment shows that residual risks—i.e. additional risks compared to today that remain despite adaptation; see SM2—are to be expected even in the most favourable combination of ambitious climate mitigation and high adaptation (Fig. 3C). For Urban Atoll Islands and Arctic Communities, even ambitious mitigation and adaptation efforts will result in residual risks about equal to a 20% increase in today’s risk level. Under the RCP8.5 upper likely range, residual risks will consist of almost a doubling of today’s risk level in Arctic communities (+ 94%) and more than a doubling in Urban Atoll Islands (+ 139%). Given recent estimates showing that the global mean surface temperature is rapidly approaching 1.5 °C above preindustrial levels^113^, these results suggest that today’s risk levels (*Moderate*) will be substantially exceeded over this century, and so that relatively small communities in highly SLR-sensitive ecosystems will especially be subject to adaptation limits, including broader detrimental cascading effects on lives, livelihoods and identities.

The situation appears a priori less problematic for Large Tropical Agricultural Deltas, where end-century residual risks could be limited to about *Moderate* even under RCP8.5 median (24% on the *Undetectable*-*Extremely high* risk scale compared to 16% today). However, while a RCP2.6-High adaptation combination could help limiting residual risks to almost zero compared to today’s risk level, warmer scenarios will lead to increased residual risks by 50% under mean RCP8.5 and more than a doubling under RCP8.5 upper likely range (+ 125%). In addition, these result only consider the climate-related component of SLR projections, therefore leaving aside the potentially substantial contribution of increased human-induced subsidence to future SLR at specific sites^49^, as shown for the GBM under a warming scenario higher than RCP2.6 (i.e. RCP4.5)^86^. Thus, our assessment of end-century residual risk might be considered a conservative estimate. Actual SLR risk levels might be higher still. Additional caution relates to the fact that residual risks here apply at the entire delta scale, that is, to millions of people and high population densities.

Resource-Rich Cities present a different situation. Under the high adaptation scenario, and assuming that these locations remain rich and their governance structures perform, end-century residual risk could be kept to a minimum as long as the upper likely range of RCP8.5 is not exceeded. This will however be at the expense of costly and comprehensive infrastructure, including maintenance costs over time. As for Large Agricultural Tropical Deltas, under a RCP2.6-High adaptation combination, residual risks by the end of this century could be limited to almost zero compared to today’s risk levels; but the increase under mean RCP8.5 will be lower than in deltas and be kept to 17%. However, Resource-Rich Cities implementing high adaptation will inevitably experience a doubling of today’s risk level under the RCP8.5 upper likely range. Despite that this represents a more modest increase in relative values compared to other settlement archetypes (+ 16% on the *Undetectable*-*Extremely high* risk scale compared to + 36 to + 57% for the three other archetypes), this raises serious concerns in terms of economic loss and damages, for example.

Importantly, our analysis considers averaged populations for each archetype, while individual social groups or settlements within a given coastal archetype are heterogenous and might experience adaptation limits earlier than others^114^. This calls for both more attention to be paid to the distribution of residual risks over populations and to related justice considerations in local adaptation planning.

## Unresolved questions and future directions

This work raises four important challenges for the assessment of the additional risks induced by future SLR and adaptation efforts.

First, there is a need to include (1) more comprehensive SLR scenarios, (2) local socioeconomic scenarios, and (3) more diversified adaptation scenarios. On SLR (1), while our assessment does include estimates of changes in extreme water levels at the coast driven by regional SLR only, it does not explicitly quantify extreme event climatology or changes therein^48^, such as a change in tropical cyclone frequency. This results in the potential underestimation of future risk levels. In addition, it would make sense to include approaches other than SLR estimates from process-based models alone, such as expert elicitation, despite lower confidence in the associated estimates. Decision-makers with a low risk tolerance (e.g., planning for long-term investment in infrastructure) might indeed rely on such estimates for the assessment of low probability outcomes outside the RCPs’ likely range^17^, e.g. 2 m in 2100 under a high emission scenario^115^. On socioeconomic scenarios (2), Shared Socioeconomic Pathways (SSPs) provide a global-scale perspective on future societal conditions related to trends in, for example, demographics, economics, and governance^116^. They can be used to combine future trajectories in socio-economic exposure and vulnerability with climate hazards, as done for instance in the recent IPCC Special Report on *Climate Change and Land*^117^, and therefore assess the dynamics of future risk in a more comprehensive way. However, studies downscaling the SSPs at a regional or local level are only emerging^118,119^ and no robust approach has been proposed to date that allow for consistent analysis of socioeconomic trajectories for a wide diversity of particular locations. On adaptation (3), while considering the two ends of the adaptation spectrum (none-to-moderate vs. high) allows emphasizing the range of potential benefits, intermediate adaptation scenarios have the same probability of realization, if not higher. Intermediate scenarios however call for including a wide range of context-specific barriers to adaptation (e.g., social, cultural, institutional, economic)^50,72^ for which assessing the contribution to risk reduction remains highly challenging.

Second, our assessment only considers present-day and end-century time slices, implying a roughly linear development of risk over the century (see curve shaping in Fig. 3C). Climate change effects on SLR and extreme events however have the potential to accumulate in compound events^120^, cause cascading impacts, and result in tipping points^4,13^. SLR also is expected^6^ to accelerate globally to a mean rate of 15 mm/year by 2100 under RCP8.5. In addition, thresholds in social-ecological systems (e.g. shift from reefs to microalgae; or when policies are rendered untenable due to climate change or changing conditions and are perceived as undesirable by societies^121^) must be considered that will affect a given settlement’s vulnerability and adaptive capacity to SLR. Overall, risk development will likely not be quasilinear but rather potentially exponential with possible intermittent jumps. Capturing such complexity will help improve knowledge on end-century adaptation benefits in terms of both risk reduction and the time we could potentially buy through ambitious adaptation (i.e. risk delay^4^).

Third, there is a need to better understand the implications of the long-term commitment to SLR for adaptation strategies and targets. It is virtually certain that sea level will continue to rise beyond 2100, if only because of the slow response time of the ocean and ice sheets. Values for 2300—though associated with low confidence—range from 0.3–3.1 m to 1.7–6.8 m for low and high emission scenarios, respectively^43^. In addition to raising the challenge of urgently operationalizing highly ambitious mitigation efforts^13^, the long-term commitment to SLR highlights the role of ambitious twenty-first century adaptation in laying the foundations for post-2100 risk reduction.

Last, the needs to consider more comprehensive sea level change scenarios, the non-linearity of risk dynamics and the long-term commitment to SLR, call for expanding the IPCC risk scale by adding a new threshold for *Extremely high* risk, as done in this study. This level is not reached in our assessment because our study focuses on the additional contribution to risk from SLR only and within the twenty-first century, leaving aside other risk factors (changes in extremes, for example) and longer timescales. It is however likely that a more comprehensive assessment framework would have led to reaching *Extremely high* risk levels, for example for Urban Atoll Islands under the upper likely range of RCP8.5. In that view, the transition from *Very High* to *Extremely High* risk could provide a useful space to discuss the limits to adaptation, both locally and globally^14^, and strengthen the connections with research on “severe climate risk”^122^, existential risk^54^ and catastrophic risk^55^.

## Methods

This “Methods” section presents the methodological foundations of the formal expert-judgement exercise to assess the additional coastal risk induced by sea level rise (SLR) and the benefits of adaptation for risk reduction. It is inspired from the “Supplementary Material S1” provided with chapter 4 of the SROCC *(16)* (available here: https://www.ipcc.ch/site/assets/uploads/sites/3/2019/11/SROCC_Ch04-SM_FINAL.pdf). However, new literature has been considered since the SROCC and a more quantitative analysis has been developed specifically for this study (see sub-section “Relative risk scores” below). The final results, which have been used as background material to develop Figs. 3 and 4 (which are also new compared to the SROCC), are synthesized in Table 2 and detailed in Supplementary Material SM1 (scoring system) and SM2 (detailed results).

### Overview

**Figure 5:**
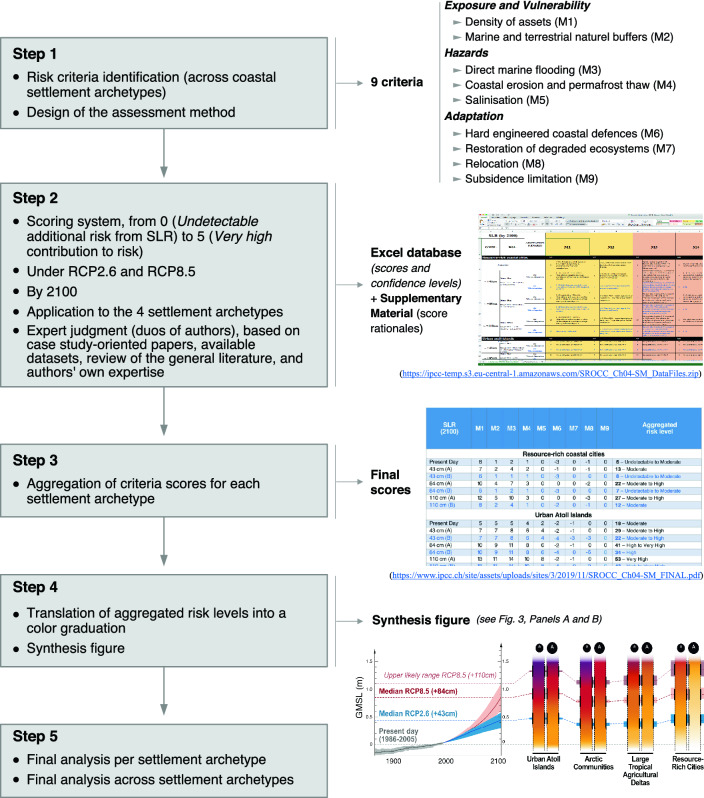
Overview of the methodological protocol for the SLR risk assessment.

The risk assessment relies on the expert judgment of the authors of this paper, who all contributed to the SLR chapter of the SROCC^1^. Due to knowledge gaps inherent to the absence of systematic and standardized sea level and coastal impact projections at the specific sites considered under each of the settlement archetypes, the authors used formal expert judgement as alternative approach to understand the additional coastal risk induced by SLR (Box 1). The formal expert judgment relies on (1) an evaluation of the peer-reviewed literature; (2) the 10-to-50-year experience of the authors in SLR-related risks in the coastal settlement archetypes considered; (3) a matrix composed of nine risk criteria; and (4) a scoring system to characterize risk levels. The assessment followed a 5-step approach (Fig. 5) and has been applied to the coastal archetypes described in Box 1 and in Table 1.

### Risk criteria (step 1 in Fig. 5)

The assessment matrix is composed of nine prominent criteria—called metrics (M) in the SROCC—contributing to the additional coastal risks induced by SLR locally, and reflecting the main components of risk, i.e. exposure and vulnerability, hazard, and adaptation responses (Table 3 below).

- The ***density of assets*** at the coast (M1) and the degree of degradation of natural buffer ecosystems (M2; e.g. mangroves, beach-dune systems) serve as proxies for the socioecological system’s exposure and vulnerability to SLR, and illustrate some of the prevailing anthropogenic drivers of risk described in the “Non-climatic anthropogenic drivers of risk” section of the main manuscript. Despite the importance of considering socioeconomic scenarios in future-oriented risk assessment, the analysis needed to assume relatively stable coastal population density over the century due a lack of consistent population and socioeconomic scenarios across the settlement archetypes and case studies considered.
- Three indicators provide proxies of the three ***main hazards*** depicted in the “Range of impacts” section of the main manuscript: direct marine flooding (M3), coastal erosion or permafrost thaw (M4), and salinization of groundwater lenses, soils and surface waters (M5).
- Four indicators describe the ***range of potential adaptation measures***^88^: the implementation of hard-engineered coastal defences (M6); the restoration of degraded ecosystems and/or creation of new natural buffer areas (M7); the planned and local-scale relocation of people, assets and activities (excluding forced displacement and international migration) (M8); and the limitation of human-induced subsidence especially in deltas and coastal cities (and assuming continuation of current subsidence trends; M9).

### Sea level rise markers by the end of the century

This paper considers ***RCP2.6 and RCP8.5*** because, first, these scenarios allow to illustrate the wide spectrum of SLR and associated risks, and although we recognize that current global emissions are more closely aligned^123^ with intermediate scenarios RCP4.5 and RCP6.0 —though underestimation issues^124^. The RCP8.5 scenario has been criticized^123^ for being a misleading warming scenario in that it is widely associated with a “business-as-usual” greenhouse gas emission trajectory, while it has originally been designed to describe an unlikely high-risk future. By using RCP8.5, we do not assume it to be likely or unlikely, or even realistic or unrealistic, but simply use it to expand the scope of this analysis. Second, there is still a gap in the systematic use of intermediate scenarios in the impact-oriented literature and for all the topics and coastal archetypes addressed in this paper, while risks under RCP2.6 and RCP8.5 are far better covered.

This assessment focuses on the ***additional risks due to SLR*** and does not explicitly quantify changes in extreme event climatology (waves, cyclones, etc.). Based on this framing, the analysis uses Global Mean Sea Level rise (GMSL) by 2100 relative to Present Day (1986–2005) as a representation of different possible climate change scenarios (see Panel A in Fig. 3). Three GMSL levels are considered as re-assessed in the SROCC: + 43 cm for mean RCP2.6 (range 0.29–0.59 m); + 84 cm for mean RCP8.5 (range 0.61–1.10 m), and + 110 cm for the RCP8.5 upper end of the likely range. The assessment of additional risks due to SLR on specific settlements is however not developed directly against GMSL, but against end-century regional mean sea level (RSL) rise in order to allow a locally accurate approach.

***Regional sea level projections*** (see section “Projections of SLR” in the main manuscript) are used to describe RSL rise for the different real-world case studies, and then have been averaged at the respective archetype level (see Table 1 for details, and background information in Panel B of Fig. 3). For example, RSL projections for Rotterdam or Shanghai are based on the broader situation of the Dutch delta and the Yangtze Estuary, respectively. However, as the nine risk indicators considered in our assessment rather reflect more local contexts, they describe the risk situation of (e.g.) Rotterdam and Shanghai themselves but not coastal risks at the broader scale of the Dutch delta and the Yangtze Estuary, respectively. This framing applies to the four archetypes: Resource-rich coastal cities, Arctic communities (focus on specific ones, but not whole regions), Urban atoll islands (focus on specific islands, not the whole atoll or regional level), and Large agricultural tropical deltas (focus on portions that are a significant fraction of the entire country, i.e. Vietnam and Bangladesh, but not broader regional, multi-country level).

### Human-induced subsidence

To be able to consider natural and human-induced subsidence in our risk assessment, and in the absence of robust information on future human-induced changes in subsidence rates at the study locations, we hypothesized the continuation of current trends^46^. The consideration of subsidence justifies the adaptation criterion referring to “Limiting subsidence” (M9).

### Adaptation scenarios

In order to capture the range of potential risk reduction from adaptation, two scenarios have been considered that illustrate the two ends of the adaptation spectrum:

- “***None-to-moderate adaptation***” represents a business-as-usual scenario where no major additional adaptation efforts compared to today are implemented. That is, neither substantial intensification of current actions nor new types of actions; for example, only moderate raising of existing protections in high density areas or sporadic episodes of coastal relocation or beach nourishment where large-scale efforts are not already underway.
- “***High adaptation***” represents the opposite situation, that is, an ambitious combination of both incremental and transformational adaptation that leads to significant additional efforts compared to today. Examples of measures are the relocation of entire districts in a megacity or the creation or restoration of beach-dune systems at a significant scale. This scenario assumes that adaptation efforts are implemented at their full potential, that is, the extent of adaptation that is technologically possible, with minimal financial, social and political barriers. This framing of course applies differently in the various settlement archetypes considered in this study, as the technological feasibility of hard coastal protection (M6), ecosystem-based adaptation (M7) and planned local relocation (M8), for example, is highly context-specific.

### Scoring system (steps 2 and 3 in Fig. 5)

Based on the IPCC Reasons for Concern framing, seven coastal risk levels have been considered: *Undetectable*, *Undetectable-to-moderate*, *Moderate*, *Moderate-to-high*, *High*, *High-to-very high*, and *Very high*. At the indicator (M) stage first, scores have been associated to each risk level, ranging from 0 to 6 (from *Undetectable* to *Very high*, and assuming a linear scale), in order to reflect the additional contribution of SLR to coastal risk for a given indicator. A first round of expert judgment consisted of assigning, for a given archetype, a risk score to each of the indicators (M1–M9) using insights from the real-world case studies as well as additional literature. Positive and negative scores have been respectively assigned to M1–M5 and M6–M9 to describe contributions to increasing or decreasing risk, respectively. This allowed for describing the Present-day coastal risk at the archetype-level (scores normalized and aggregated using equal weights). Based on Present-day risk scores, the future additional contribution of SLR to coastal risk have been assessed for each indicator at each of the end-century SLR markers (+ 43 cm, + 84 cm, + 110 cm in GMSL) following this three-fold approach:

1. Additional scores of + 1, + 2 and + 3 have been applied to indicators M1–M5 to estimate a respectively low, substantial and very substantial contribution to increasing risk by the end-century.
2. Additional scores of − 1, − 2 and − 3 have been applied to indicators M6-M9 to estimate a respectively low, substantial and very substantial contribution to decreasing risk by the end-century.
3. To reflect some degree of cumulative risk as sea level rises, scores for the + 43 cm SLR scenario built on Present-day scores, when scores for the + 84 cm and + 110 cm SLR scenarios built on the + 43 cm and + 84 cm scores, respectively.

The aggregation (addition, no weighting) of M1–M5 scores (1) describes the increase in additional risk induced by SLR at the coastal archetype level, for each SLR scenario and under a “none-to-moderate” adaptation scenario. The additional consideration of M6–M9 scores (2) provides the assessment of risk under a “high adaptation” scenario, and for the different SLR markers.

### Relative risk scores

In order to move the SROCC analysis a step further, we estimated the relative risk scores against the full range of possible scores (from *Undetectable* to *Extremely High*). In that view, we rescaled the absolute aggregated risk scores along a 0–100% scale. Calculation formula are presented in cells G1–Q3 in SM2 and results are shown in columns W and X. This allowed for comparing risk increase across SLR scenarios, as well as calculating the relative decrease in risk levels associated with various adaptation scenarios (column Y) compared to Present-day risk level. The same approach was applied to the quantitative description of residual risk levels (column Z in SM2).

### From risk scores to burning embers (Fig. 3, step 4 in Fig. 5)

The archetype-level aggregated risk scores have then been translated into the IPCC colour scale used to develop the burning ember diagrams and that goes from white (*Undetectable* risk) to yellow, orange, red and purple (*Very high* risk). Important, for the end-century, we added a new risk category to the IPCC framing in order to reflect the potential accumulation of maximum risk scores in all of the indicators, hence giving an indication of a very high-end risk scenario above which hard limits to adaptation may widely occur—i.e. limits that cannot be overcome, as opposed to “soft” limits^125^. The establishment of this equivalence between risk levels and colours laid the foundations for developing Fig. 3 (see Panel B and legend), based on the following framing.

- For ***Present-day risk levels***, with aggregated scores ranging from 0 to 30, seven equidistant classes have been established: 0 (*Undetectable* contribution to risk for all indicators; white), 5 (*Undetectable-to-moderate*; white to yellow), 10 (*Moderate*; yellow), 15 (*Moderate-to-high*; orange), 20 (*High*; red), 25 (*High-to-very high*; red to purple), and 30 (*Very high*; purple);
- For ***End-century risk levels***, with aggregated scores ranging from 0 to 75, nine equidistant classes have been established: 0 (*Undetectable* contribution to risk for all metrics; white), 9–10 (*Undetectable-to-moderate*; white to yellow), 18–19 (*Moderate*; yellow), 28–29 (*Moderate-to-high*; orange), 37–38 (*High*; red), 46–47 (*High-to-very high*; red to purple), 56–57 (*Very high*; purple), 65–66 (*Very high-to-extremely high*; purple to dark purple), and 75 (*Extremely high*; dark purple).

### Development of the synthesis figures

The results presented in Table 2 in the main manuscript (and SM2) served as foundations to develop two synthesis figures:

- *Figure **3* is Burning embers in (B) are reproduced from the IPCC SROCC *(17)*. Panel C displays the assessment results (aggregated scores) against the *Undetectable-Extremely high* risk scale represented by the vertical white-to-dark purple bars on the right-hand side of each of the settlement archetype plot. The positioning of the end-century risk levels under various SLR and adaptation scenarios—right-hand side of the curves—is informed by the aggregated scores and their location along the *Undetectable-Extremely high* risk scale (0–75; SM2), and reflect the results used to develop the burning embers in (B).
- *Figure **4* is The right-hand side panel uses the same material as Fig. 3 but displays results against a horizontal risk gradation (white-to-dark purple background) instead of a vertical risk graduation as in Fig. 3B,C.

## Supplementary Information

Supplementary Information.

## Data Availability

All data generated or analysed during this study are included in this published article and its supplementary information file. All figures have been drafted by the authors especially for this article.
